# Endothelial Dysfunction of Bypass Graft: Direct Comparison of *In Vitro* and *In Vivo* Models of Ischemia-Reperfusion Injury

**DOI:** 10.1371/journal.pone.0124025

**Published:** 2015-04-15

**Authors:** Gábor Veres, Péter Hegedűs, Enikő Barnucz, Raphael Zöller, Stephanie Klein, Harald Schmidt, Tamás Radovits, Sevil Korkmaz, Matthias Karck, Gábor Szabó

**Affiliations:** 1 Department of Cardiac Surgery, University of Heidelberg, Heidelberg, Germany; 2 Heart Center, Semmelweis University, Budapest, Hungary; Thomas Jefferson University, UNITED STATES

## Abstract

**Background:**

Although, ischemia/reperfusion induced vascular dysfunction has been widely described, no comparative study of in vivo- and in vitro-models exist. In this study, we provide a direct comparison between models (A) ischemic storage and in-vitro reoxygenation (B) ischemic storage and in vitro reperfusion (C) ischemic storage and in-vivo reperfusion.

**Methods and Results:**

Aortic arches from rats were stored for 2 hours in saline. Arches were then (A) in vitro reoxygenated (B) in vitro incubated in hypochlorite for 30 minutes (C) in vivo reperfused after heterotransplantation (2, 24 hours and 7 days reperfusion). Endothelium-dependent and independent vasorelaxations were assessed in organ bath. DNA strand breaks were assessed by TUNEL-method, mRNA expressions (caspase-3, bax, bcl-2, eNOS) by quantitative real-time PCR, proteins by Western blot analysis and the expression of CD-31 by immunochemistry. Endothelium-dependent maximal relaxation was drastically reduced in the in-vivo models compared to ischemic storage and in-vitro reperfusion group, and no difference showed between ischemic storage and control group. CD31-staining showed significantly lower endothelium surface ratio in-vivo, which correlated with TUNEL-positive ratio. Increased mRNA and protein levels of pro- and anti-apoptotic gens indicated a significantly higher damage in the in-vivo models.

**Conclusion:**

Even short-period of ischemia induces severe endothelial damage (in-vivo reperfusion model). In-vitro models of ischemia-reperfusion injury can be limitedly suited for reliable investigations. Time course of endothelial stunning is also described.

## Introduction

Vascular grafts are important therapeutic option for bypass surgery. The long-term benefit of bypass surgery depends largely on the long-term patency of bypass grafts, which are determined by several factors: the progress of heart/vascular disease, the run-off and the biological properties of the implanted graft, injuries during surgical manipulation and the degree of ischemia/reperfusion (IR) injury.

Regarding the importance of an intact endothelial layer for graft patency, numerous experimental animal models have been developed in an attempt to understand the developing pathophysiological processes of IR injury on the bypass graft. A simple, clinically relevant in vitro experimental model and/or an in vivo model for vascular IR injury would obviously offer great prospects in the field of vascular pharmacological research. While the complex nature of IR injury cannot possibly be replicated fully in in vitro circumstances, these animal models must provide a reproducible paradigm allowing the investigation and evaluation of endothelial dysfunction.

Throughout the years, various in vitro animal models have been utilized to mimic endothelial dysfunction: (A) In vitro model of ischemia (hypoxia-reoxygenation) [1–3] and (B) in vitro models of ischemia-reperfusion [4–7] are two dominant models of vascular injury. However, it was proved that endothelial injury occurring in vessels during in vitro hypoxia and reoxygenation is too moderate to induce functional alterations of the endothelium, probably due to lack of activated leucocytes [8]. Several recent in vitro studies used successfully the hypochlorite to mimic the detrimental effect of reperfusion injury [4–7]. However, these in vitro models could not demonstrate a severely reduced vasorelaxation function after IR injury, which is not fully correlated the results after bypass surgery published by several animal and human studies. To observe the time course of vascular injury and the in vivo effect of IR injury, we developed a new rat model of arterial revascularization.

This study aims to compare models of in vivo induced reperfusion injury of transplanted rat aortic arches with the in vitro injury models where oxidative damage is caused by reoxygenation and/or incubation in hypochlorite. Additionally, time course of endothelial dysfunction and recovery was also studied using a newly developed in vivo IR model in rat.

## Methods

### Animals

Young male Lewis rats (250 to 330 g; Charles River, Sulzfeld, Germany) (N = 5–7/group) were used for our experiments. All procedures concerning animals were conformed to the Guide for the Care and Use of Laboratory Animals prepared by the Institute of Laboratory Animal Resources and published by the National Institutes of Health (NIH Publication No. 86–23, revised 1996). The investigations were reviewed and approved by the Ethical Committee for Animal Experimentation of Semmelweis University (Permit Number: 22.1/1934/3/2011).

### Experimental groups

Control group aortic arches were cut to rings and freshly mounted for organ bath. Arches in all other groups went through 2 hours of 4°C storage in saline solution. In the ischemia group arches were then cut to rings and mounted for organ bath. Aortic rings in the 3. group following 2 hours ischemia were mounted and were incubated in 200μM HOCl for 30 min before organ bath measurements (the applied dose and timing were widely used in previous investigations with hypochlorite-induced reperfusion injury [7, 9, 10]). Aortic arches for in vivo reperfusion following the 2 hours ischemia were transplanted and reperfused for 2, 24 hours and 7 days. They were then explanted again and mounted in organ bath. The list of groups and treatments is shown on Table 1.

**Table 1:** Groups and treatments.

Groups	Ischemia	Reperfusion
(1) Control	-	-
(2) Ischemia	2h storage in saline	-
(3) Ischemia + In vitro R	2h storage in saline	30min hypochlorite incubation
(4) Ischemia + In vivo R (2h)	2h storage in saline	2h in vivo reperfusion
(5) Ischemia + In vivo R (24h)	2h storage in saline	24h in vivo reperfusion
(6) Ischemia + In vivo R (7d)	2h storage in saline	7d in vivo reperfusion

### Aortic arch preparations for in vitro experiments

Rats were euthanized by an overdose of sodium-pentobarbital before exsanguination. Aortic arches were isolated and according to the experimental groups (control and in vitro groups) transferred to cold (4°C) Krebs–Henseleit solution (118 mmol/L NaCl, 4.7 mmol/L KCl, 1.2 mmol/L KH_2_PO_4_, 1.2 mmol/L MgSO_4_, 1.77 mmol/L CaCl_2_, 25 mmol/L NaHCO_3_, and 11.4 mmol/L glucose; pH = 7.4), physiological saline solution respectively, cleaned of superficial and loose connective tissue. Special attention was paid during the preparation to avoid damaging the endothelium. Aortic arches of the control group went through no cold ischemic storage, were cut to 4 mm rings and mounted after preparation in organ bath, and received no hypochlorite exposition. Aortic arches, excluded control group were cut to 4 mm pieces and stored in 4°C for 2 hours in 5ml cold saline containing tubes. Previously the tubes were equilibrated for 15 minutes with nitrous oxide to extrude oxygen from the solutions. After 2 hours of cold ischemic storage, rings were mounted to organ bath.

### Aortic arch transplantation for in vivo experiments

Aortic transplantations were performed in isogeneic Lewis to Lewis rat strain, therefore no organ rejection can be expected. The rats were anaesthetized with a mixture of ketamine (100 mg·kg-1) and xylazine (3 mg·kg-1) intraperitoneally, tracheotomized and intubated to facilitate breathing. The animals were placed on controlled heating pads, and core temperature measured via a rectal probe was maintained at 37°C. Intraoperatively, the depth of anaesthesia was assessed using the toe pinch method. As analgesia, 0.05.mg/kg buprenorphin was injected subcutaneously twice daily in the first three postoperative days. Aortic arches were isolated and transferred to cold (4°C) physiological saline solution. Explanted aortic arches were stored at 4°C for 2 hours in 5ml cold saline containing tubes. Previously the tubes were equilibrated for 15 minutes with nitrous oxide to extrude oxygen from the solutions. After the 2 hours ischemia, systemic anticoagulation was performed in the recipient rat and the aortic arch was heterotopically transplanted by two end-to-end anastomoses into abdominal aorta of the isogenic recipient. Two or 24 hours or 7 days after transplantation, animals were sacrificed with an overdose of sodium pentobarbital (150 mg/kg, intraperitoneally) and the implanted graft is harvested. Graft segments were cut transversely into 4-mm width rings. Isolated aortic rings were mounted on hooks in individual organ baths (Radnoti Glass Technology, Monrovia, CA, USA).

### *In vitro* organ bath experiments

Isolated rings were mounted on stainless steel hooks in individual baths containing 25 ml of Krebs–Henseleit solution at 37°C and aerated with 95% O_2_ and 5% CO_2_. Isometric force was recorded through force transducers (159901A, Radnoti Glass Technology, Monrovia, CA, USA), with IOX Software System (EMKA Technologies, Paris, France). Resting tension was adjusted to 20mN (found optimal in preliminary experiments [11, 12]) and the rings were allowed to equilibrate for at least 60 min. At 30 min intervals the medium was exchanged for fresh buffer. After equilibration period maximal contraction forces to potassium chloride (KCl, 80 mM) were determined and aortic rings were washed until the resting tension was again obtained.

To stimulate free radical burst 200 μM hypochlorite was added for 30 minutes to the baths of in vitro group of IR for modeling reperfusion, then washed out. Aortic preparations were preconstricted with phenylephrine (10^− 6^ M) until a stable plateau was reached and relaxation responses were examined by adding cumulative concentrations of endothelium-dependent dilator acetylcholine (10^− 9^–10^− 4^ M). For testing relaxing response of smooth muscle cells, a direct nitric oxide donor, sodium nitroprusside (10^− 10^–10^− 5^ M) was used. Half-maximal response (EC_50_) values were obtained from individual concentration–response curves by fitting experimental data to a sigmoidal equation using Origin 7.0 (Microcal Software, Northampton, USA). Contractile responses to phenylephrine are expressed as percent of the maximal contraction induced by KCl. The sensitivity to vasorelaxants was assessed by pD_2_ = − log EC_50_ (M), vasorelaxation (and its maximum (*R*_max_)) is expressed as percent of the contraction induced by phenylephrine (10^− 6^ M).

### Histopathologial processing

Aortic segments from each experimental group were fixed in buffered paraformaldehyde solution (4%) and embedded in paraffin. Then, 4-μm-thick sections were placed on adhesive slides.

### CD31 immunhistochemical staining

To detect the loss of endothelial cells in the lumen of the aortic arches, CD31 staining was performed. The endothel-covered area was measured by Cell^A imaging software (Olympus, Hamburg, Germany). During confocal analysis, the vessel was imaged. Endothelium covered areas of the vessels were demarcated on the screen by manual tracking and the re-endothelisation expressed as a percentage of the lumen area. The evaluation was conducted by an investigator blinded to the experimental groups.

### Terminal deoxynucleotidyl transferase-mediated dUTP nick-end labeling (TUNEL) reaction

To detect DNA strand breaks TUNEL assay was performed. Following the protocol of the commercial kit provided by the manufacturer (Roche Diagnostics, Mannheim, Germany) the sections were incubated with 50 μl of Terminal deoxynucleotidyl Transferase (TdT) enzyme and TUNEL Reaction mixture for 1 h at 37°C in the dark. The sections were then washed with PBS (1×) for 3×5 min. The slides were mounted using 4′, 6-diamidino-2-phenylindole (DAPI)-Fluoromount-G (SouthernBiotech, Birmingham, USA), covered with cover glass and analyzed under a fluorescence microscope. The number of TUNEL-positive cells was expressed as the ratio of DAPI-TUNEL double-labeled nuclei to the total number of nuclei stained with DAPI. Cells were counted in four fields characterizing each specimen, and an average value was calculated for each experimental group. The evaluation was conducted by an investigator blinded to the experimental groups.

### Quantitative Real-Time Polymerase Chain Reaction (rtPCR)

Total RNA was isolated from the chosen aortic rings with RNeasy Fibrous Tissue Mini Kit (Qiagen, Hilden, Germany) after homogenisation. RNA concentration and purity were determined at 260, 280, and 230 nm wave-length with. Reverse transcription was performed with the QuantiTect Reverse Transcription Kit (Qiagen, Hilden, Germany) using 400 μg RNA in a volume of 20 μL. Quantitative real-time PCR was performed on the LightCycler480 system with the LightCycler480 Probes Master and Universal ProbeLibrary probes (Roche, Mannheim, Germany). Efficiency of the PCR reaction was confirmed with standard curve analyis. Every sample was quantified in duplicate, normalized to glyceraldehyde-3-phosphate dehydrogenase (GAPDH) expression. Primers were obtained from TIB Molbiol (Berlin, Germany), their sequences and UPL probes used are represented on Table 2. Evaluation was performed with LightCycler 480 SW1.5 software (Roche, Mannheim, Germany).

**Table 2:** The sequences for the forward (F) and reverse (R) primers (from 5’ to 3’) and Universal Probe Library (UPL) probes. eNOS: endothelial nitric oxide synthase; GAPDH: glyceraldehyde-3-phosphate dehydrogenase.

Assay	Sequence	UPL probes
Bax	F: 5’-TAGCAAACTGGTGCTCAAGG, R: 5’-GCCACCCTGGTCTTGGAT	69
Bcl-2	F: 5’-GTACCTGAACCGGCATCTG, R: 5’-GGGGCCATATAGTTCCACAA	75
Caspase-3	F: 5’-AAACCTCCGTGGATTCAAAA, R: 5’-AGCCCATTTCAGGGTAATCC	56
eNOS	F: 5’-TGACCCTCACCGATACAACA, R: 5’-CGGGTGTCTAGATCCATGC	5
GAPDH	F: 5’-CTACCCACGGCAAGTTCAAT, R: 5’-ATTTGATGTTAGCGGGATCG	111

### Western blotting

Proteins were extracted from tissue homogenates and Western blotting was perfomed for the quantification of caspase-3, Bax, eNOS and Bcl-2 fragment. Glyceraldehid-3-phopsphate dehydrogenase was determined as housekeeping protein (Santa Cruz Biotechnology, Heidelberg, Germany). Target protein densities were normalized to housekeeping densities of the same sample.

### Chemical Reagents

Sodium-hypochlorite solution was produced by Grüssing, Filsum, Germany. For anesthetic sodium-phenobarbital (MerialGmbH, Hallbergmoos, Germany) was used. Phenylephrin, acetylcholine and sodium nitroprusside were obtained from Sigma-Aldrich (Taufkirchen, Germany).

### Statistical Analysis

Data were tested for normal distribution with Shapiro-Wilk and expressed as the means±SEM. Statistical significance was determined by ANOVA followed by Bonferroni multiple comparison post hoc test. P<0.05 was considered statistically significant.

## Results

### Vasomotor function

**Fig 1:**
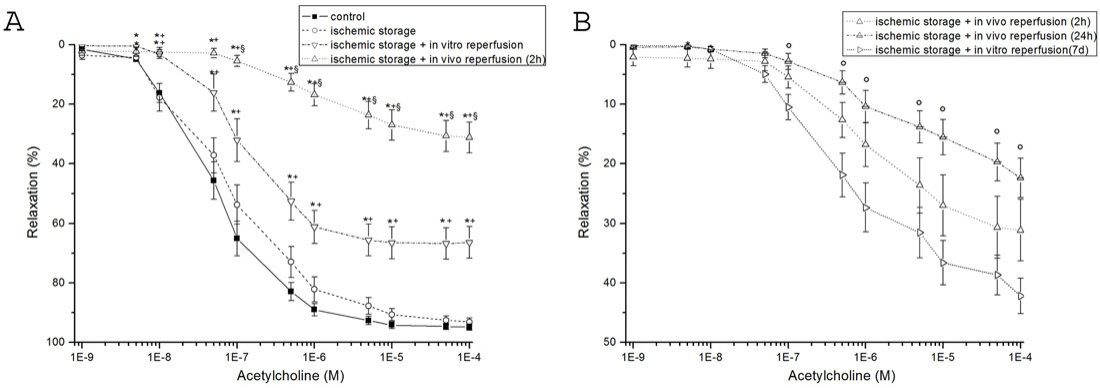
Vasomotor function of rat aortic rings. (A) acetylcholine-induced endothelium-dependent vasorelaxation are shown in control, ischemic storage, ischemic storage and in vitro reperfusion, ischemic storage and in vivo 2h reperfusion groups. (B) acetylcholine-induced endothelium-dependent vasorelaxation are shown in control and ischemic storage and in vitro reperfusion groups. Values represent mean ± SEM; *P<.05 versus control; +P<.05 versus ischemic storage; §P<.05 versus ischemic storage and in vitro reperfusion; ° p < 0.05 vs. ischemic storage and in vivo 7d reperfusion.

In aortic rings precontracted with PE, ACh induced a concentration-dependent relaxation (Fig 1A). Ischemic storage and in vitro reoxygenation had not effect on endothelium-dependent vasorelaxation, however a marked, significant decrease was shown in the ischemic storage and in vitro reperfusion group when compared to the control (Fig 1A). After short ischemia and 2 hours of warm reperfusion, a significant impairment of endothelial function of arterial rings was demonstrated as compared to the control and in vitro groups. The endothelial dysfunction was indicated by the reduced maximal relaxation of aortic rings to ACh and the rightward shift of the concentration–response curve as compared with control (Fig 1). After 24 hours of warm reperfusion, the endothelial function worsened compared to the 2 hours reperfusion group without reaching the level of statistical significance (Fig 1B). Partially recovered endothelial function was observed after 7 days of warm reperfusion (Fig 1B). There was no significant difference in Rmax for endothelium-independent vasorelaxation of the aortic rings to SNP between the experimental groups (Table 3). Contractile responses of aortic rings to PE (10^-6^ M) are shown in Table 3. Significantly increased PE-induced maximum contraction was shown in the *in vitro and in vivo groups* compared to the control group (Table 3).

**Table 3:** Values of maximal relaxation (R_max_,%) and pD_2_ to acetylcholine (ACh), to sodium nitroprusside (SNP) and contraction forces induced by phenylephrine (10^-6^M) in aortic rings in control, ischemic storage, ischemic storage and in vitro reperfusion, ischemic storage and in vivo 2h reperfusion, ischemic storage and in vivo 24h reperfusion, ischemic storage and in vivo 7d reperfusion group.

	Control	Ischemic storage	Ischemic storage + in vitro reperfusion	Ischemic storage + in vivo 2h reperfusion	Ischemic storage + in vivo 24h reperfusion	Ischemic storage + in vivo 7d reperfusion
R_max_ to ACh (%)	94,9±1,1	93,1±1,4	66,4±5,3^*a*^ ^,^ ^*b*^ ^,^ ^*e*^	31,2±5,2^*a*^ ^,^ ^*b*^ ^,^ ^*c*^	22,4±3,3^*a*^ ^,^ ^*b*^ ^,^ ^*c*^	42,2 ±3,0^*a*^ ^,^ ^*b*^ ^,^ ^*c*^ ^,^ ^*e*^
pD_2_ to ACh	7,3±0,1	7,2±0,1	6,9±0,1	5,9±0,2^*a*^ ^,^ ^*b*^ ^,^ ^*c*^	5,2±0,3^*a*^ ^,^ ^*b*^ ^,^ ^*c*^ ^,^ ^*d*^ ^,^ ^*f*^	5,9±0,4^*a*^ ^,^ ^*b*^
R_max_ to SNP (%)	100,5±0,2	102,5±1,1	99,5±1,6	100,9±1,0	98,7±1,3	102,0±2,2
pD_2_ to SNP	8,2±0,1	7,9 ±0,1	7,5±0,1	7,9±0,2	7,7±0,1	7,6±0,1
Phenylephrine (% of KCl)	86,4±4,3	95,4±4,4	115,6±4,9^*a*^	118,3±3,6^*a*^ ^,^ ^*b*^	117±6,5^*a*^	176,3±8,5^*a*^ ^,^ ^*b*^ ^,^ ^*c*^ ^,^ ^*d*^ ^,^ ^*e*^

Values represent mean ± SEM

^*a*^ P<.05 versus control

^*b*^ P<.05 versus ischemic storage

^*c*^ P<.05 versus ischemic storage + in vitro reperfusion

^*d*^ P<.05 versus ischemic storage + in vivo 2h reperfusion

^*e*^ P<.05 versus ischemic storage + in vivo 24h reperfusion

^*f*^ P<.05 versus ischemic storage + in vivo 7d reperfusion.

### Expression of CD-31

**Fig 2:**
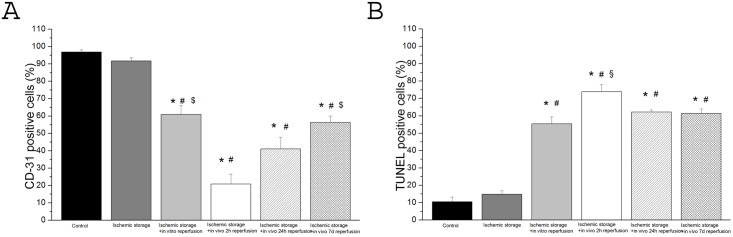
DNA strand breaks and CD-31 positive cells in aortic rings. (A) endothelium-covered area (in percentage of the lumen area) (B) scoring of TUNEL staining (in percentage of total cell number) (Magnification x200, bar = 50μm) Values represent mean ± SEM; *P<.05 versus control; #P<.05 versus ischemic storage; §P<.05 versus ischemic storage and in vitro reperfusion; $P<.05 versus ischemic storage and in vivo 2h reperfusion.

The inner walls of all aortic segments (control+other groups) were covered with endothelium CD-31 presence was detected. The percentage of stained endothelium differed between the control and the other groups. In all groups, there were regions of endothelium, which showed no reaction. In the control group the endothelial surface showing positive reaction for the CD- 31 antigen was estimated as 96±1%, whereas vessels exposed to ischemic storage it was 91±4%, to ischemic storage and hypochlorite-induced reperfusion: 61±11% (Fig 2A). After 2h in vivo reperfusion CD-31 reaction was 21±6% and after 24h warm reperfusion: 41±7% (Fig 2A). However, after 7 days in vivo reperfusion, the CD-31 reaction was significantly stronger (58±5%) as compared to the 2h reperfusion group (Fig 2A).

### TUNEL-positive nuclei

An increased density of TUNEL-positive nuclei indicates severe DNA fragmentation in the aortic wall (intima and media) of rings in all groups. In vitro/in vivo-induced reperfusion significantly increased DNA strand breaks (Fig 2B) as compared to the control.

### *In vivo* induced reperfusion injury caused pronounced changes of the mRNA expressions and protein levels

**Fig 3:**
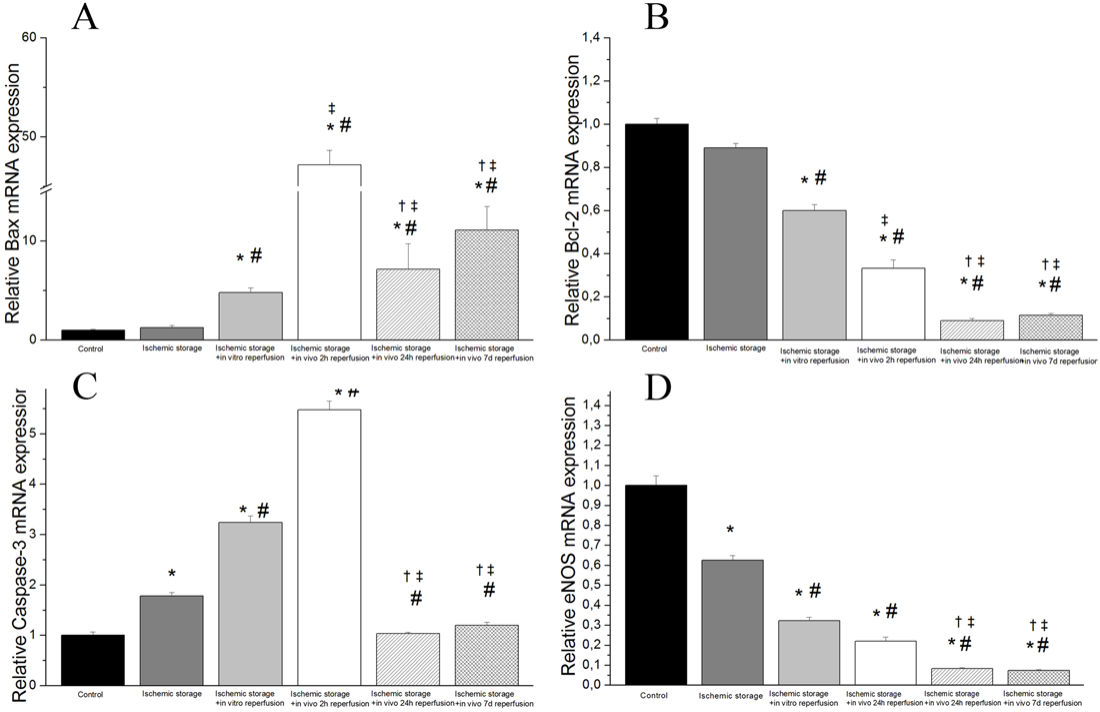
Gene expressions of (A) Bax, (B) Bcl-2, (C) caspase-3 and (D) endothelial nitric oxide synthase (eNOS) in aortic rings. Controls were given the arbitrary value of 1. Values represent median ± quartiles; *P<.05 versus control; #P<.05 versus ischemic storage; ‡P<.05 versus ischemic storage and in vitro reperfusion; †P<.05 versus ischemic storage and in vivo 2h reperfusion.

We observed a statistically relevant up-regulation in mRNA expression of two apoptotic genes caspase-3 and bax in the ischemic storage and in vitro reperfusion group and in the ischemic storage and in vivo reperfusion groups (after 2, 24 hours and 7 days reperfusion) as compare to the control (Fig 3). Furthermore, there was statistically significance up-regulation of these gene expressions in the ischemic storage and in vivo reperfusion groups when compared to the ischemic storage and in vitro group (Fig 3). We also found a significant down-regulated mRNA expression of bcl-2 and eNOS in the in vitro R and in vivo R groups (after 2, 24 hours and 7 days reperfusion) compared to control (Fig 3).

**Fig 4:**
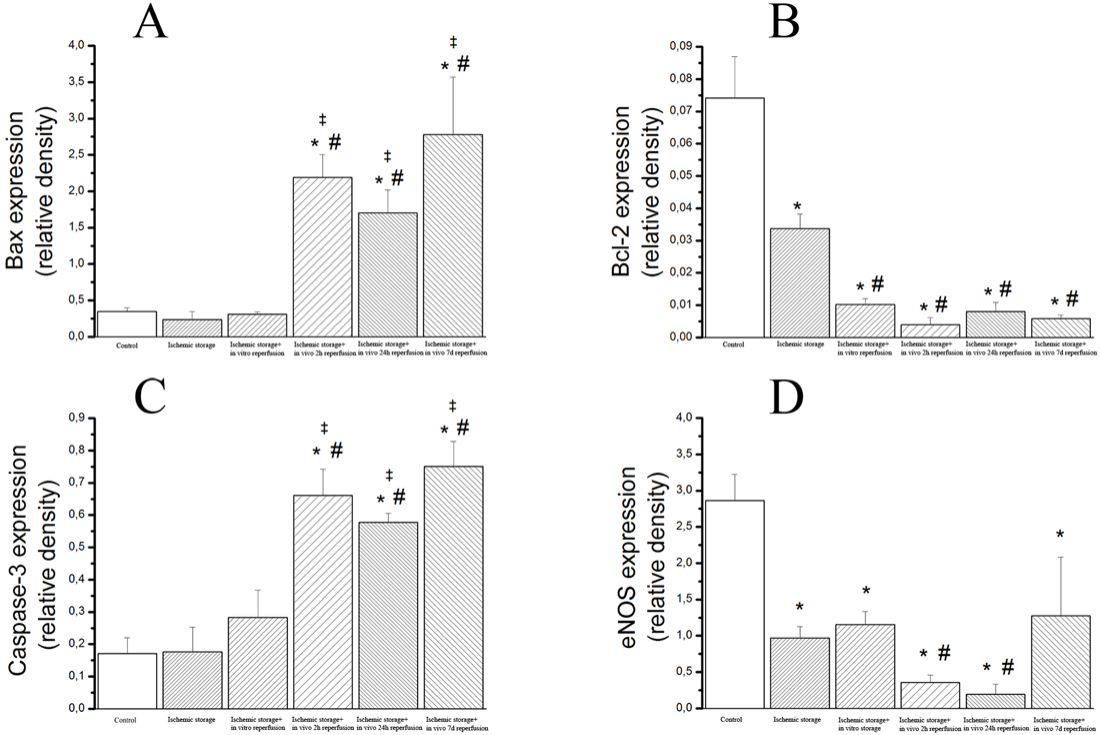
Protein expressions of (A) cleaved bax, (B) bcl-2 (C), caspase-3 and (D) eNOS in aortic rings. Values represent median ± SEM; *P<.05 versus control; #P<.05 versus ischemic storage; ‡P<.05 versus ischemic storage and in vitro reperfusion.

Densitometric analysis of caspase-3 cleavage and Bax bands showed a significant increase in the ischemic storage and in vivo reperfusion groups (Fig 4) (after 2, 24 hours and 7 days reperfusion) as compare to the control, ischemic storage and ischemic storage and in vitro reperfusion groups (Fig 4). Furthermore, we detected significantly reduced bcl-2 and eNOS protein levels in in the in vitro R and in vivo R groups (after 2, 24 hours and 7 days reperfusion) compared to control. The eNOS protein levels were also significantly reduced in the 2 and 24h in vivo reperfusion groups compared to the ischemic storage group (Fig 4). These results correlate with PCR findings.

## Discussion

Our study is the first to directly compare both *in vitro* and *in vivo* induced R injury, showing dramatically impaired endothelial function after in vivo R injury when compared to the in vitro R injury. By testing the in vitro induced R injury, we are able to detect a marked endothelial injury, however we found no endothelial impairment in the ischemic storage model.

IR injury is a complex phenomenon with serious clinical consequences and has been in focus of intensive investigations. Changes in the health of the vascular endothelium may not only have implications concerning the short-term clinical course of patients after CABG, but may affect the late patency of coronary artery bypass grafts, the progression of atherosclerosis in the native coronary circulation and the long-term success of cardiac transplants [13, 14]. The recognition that endothelial dysfunction is an early and rate-determining factor in the pathogenesis of IR injury has led to a major effort to characterize fully the vascular alterations associated with IR injury and to define the mechanisms that underlie this pathological process. In spite of the intensive investigations of this field, the exact molecular mechanisms of the pathogenesis of endothelial dysfunction of bypass graft after IR injury still remain unclear.

### Effects of ischemic storage and reoxygenation on endothelium

To date, there are only sporadic functional studies investigating in vitro vascular ischemia and reoxygenation injury [1–3, 8, 15], and the data are still remained contradictory. Isolated human endothelial cell culture investigations showed, that hypoxia and reoxygenation resulted in morphological signs of cell damage [16, 17]. A few previous investigations could show endothelial dysfunction after in vitro hypoxia and reoxygenation [2, 3, 15], but it contradict the results from other studies showing no changes in the endothelium-dependent vasorelaxation induced by ACh [1, 8]. It was also shown that prolonged (>1–2 days) cold storage is able to induce endothelial dysfunction [4, 18, 19]. In the present study we demonstrated a weak, but not significant reduction of the endothelium-dependent vasorelaxation in the vessels after 2h ischemic storage when compared to the control. Furthermore, we did not observe any difference in histopathological results (CD-31 and TUNEL) and mRNA expressions (Figs 2 and 3). Interestingly, just the eNOS mRNA expression and protein level were significantly lower in ischemic storage group when compared to the control. In agree with Radovits et al. [8], we can speculate that the endothelial damage occurred by ischemia and reoxygenation is too moderate after a clinically relevant storage time (2h), probably due to the lack of activated leukocytes and therefore is not suitable for reliable investigation of endothelial dysfunction.

### Effects of ischemic storage and in vitro induced reperfusion injury on endothelium

Ischemia and then the rapid reintroduction of molecular oxygen per se result in a burst of reactive oxygen species (ROS) during the first minutes of reperfusion [20–22]. Activated leucocytes also generate a number of ROS, including O_2_^-^, H_2_O_2_ and through the myeloperoxidase system, highly reactive hypochlorite. The luminal surface of blood vessels and vascular wall are the most exposed tissues to the deleterious effects of these reactive agents, which lead to loss of homeostatic functions of the vascular endothelium and thus capillary plugging, impaired vascular permeability, adhesion and infiltration of leucocytes [23]. Due to its role in pathophysiological conditions, such as inflammation, atherosclerosis and reperfusion injury, hypochlorite is commonly used in in vitro models of vascular I/R injury to stimulate the effect of activated leukocytes [4–7].

We showed in the present in vitro model of reperfusion injury an impaired endothelium-dependent acethylcholine-induced relaxation of aortic rings exposed to hypochlorite. The endothelium-independent vasorelaxation induced by SNP was unaffected by hypochlorite, indicating normal dilatative capacity of the vascular smooth muscle, however an enhanced PE-induced vasoconstriction was measured in this group. These functional data are consistent with the results of other studies in rabbit and rat models [6, 24, 25]. We also reported that exposure of rat aortic rings to hypochlorite resulted in formation of DNA stand breaks in the vessel wall, as evidenced by TUNEL assay. Consistently with these data, previous works on endothelial cells reported similar results [26]. Furthermore, we measured a reduction of living endothelial cells of the aortic graft, as demonstrated in the CD-31 immunohistochemical assay (Fig 2). In addition, we reported here enhanced signs of apoptosis (up-regulation of Bax and Caspase-3 levels, down-regulation of bcl-2 by PCR and Western blot experiments).

Previous in vitro models of reperfusion injury could also not observe a dramatically reduced vasorelaxation function after IR injury [4, 6, 7]. Human endothelial cell cultured experiments, however, showed that the addition of neutrophils upon reoxygenation enhances endothelial cell injury [17, 27]. The observation suggests that neutrophils become activated by some factor(s) released by endothelial cells and that the activated neutrophils exacerbate endothelial cell injury. However, experimental and clinical studies clearly showed that endothelial cells are more sensitive to IR injury than cardiomyocytes and pronounced endothelial responsiveness has also been described [28–30]. Furthermore, a human heart transplantation study clearly demonstrated that in contrast to cardiomyocytes the cell oedema of endothelium is still more pronounced after mid-term reperfusion than at the onset of ischemia [28].

We supposed that in vivo development of reperfusion injury could be the major cause of impaired short and long-term patency rate and vasospasm of the arterial graft after CABG.

### Effects of ischemic storage and in vivo induced reperfusion injury on endothelium

To observe the effect of in vivo reperfusion injury, a novel method of ischemic storage and in vivo reperfusion injury was developed. To the best of our knowledge, we demonstrated for the first time a severe endothelial dysfunction even also after 2 hour in vivo reperfusion. The peak of the endothelial damage occurred after 24 hours of reperfusion as demonstrated by dramatically damaged endothelium-dependent vasorelaxation, apoptosis rate of TUNEL-staining and increased mRNA expression of Bax and caspase-3 and the decreased levels of Bcl-2 and eNOS gene expressions. These data also correlate to the clinical findings of early complications in bypass grafts, as Lockerman and colleagues found transient ST segment elevation occuring in the first 12–24 hours after CABG [31]. Furthermore, the severity of the endothelial dysfunction (endothelium-dependent vasorelaxation and mRNA expression, protein levels, results from TUNEL and CD-31 staining) occurred by in vivo reperfusion in our model was more detrimental than in our in vitro model of IR injury.

Endothelial integrity and structure (as measured by lower expression of CD-31 on the endothelial surface in the aortic segments and enhanced DNA stand breaks) were also severely damaged in the in vivo-induced IR injury group. CD-31 is highly specific for endothelial cells and has a constant expression. After 2 hours/1 day reperfusion, a severely damaged endothelial layer of the arterial graft was also reported (Figs 1B and 4D). Surprisingly, we also showed that the function and structure of the arterial graft partially recovered after 1 week of reperfusion (Fig 1B). A previous study also reported that the endothelium is capable of regaining its functional integrity within a few days by proliferation and migration after mechanical injury [32]. Nevertheless, it was also noted, that endothelial integrity after mechanical injury could not be fully restored, since there are marked functional and structural differences between native and regenerated endothelium [33]. Regenerated endothelium exhibits impaired endothelium-dependent relaxation and signs of apoptosis. The results of our experiments are in good agreement with previous reports on the regeneration of the endothelium, as in our investigation the function of the endothelium demonstrates partial recovery after 1 week of reperfusion, however it was not fully restored (impaired vasodilatative capacity, damaged endothelial layer) and there were also signs of apoptosis (up-regulation of Bax and Caspase-3 levels, marked TUNEL-staining). The endothelial stunning in our model was also demonstrated through the dynamic changes of several genes over time, which supported the results of CD-31 staining and changed of vasomotor function over time. The fact, that relaxation to the endothelium-independent vasodilator SNP was preserved, after long-term storage and reperfusion in our model, indicated that the impaired vascular response may not be attributed to a deficit in function of vascular smooth muscle.

Much of the data derived from studies of IR-induced vascular dysfunction are consistent with the role for NO as participant in the changed endothelium-dependent responses [23]. Due to a low NO level, the endothelium-dependent vasorelaxation is compromised. We showed that the eNOS mRNA expression and protein level (Figs 3D and 4D) are drastically reduced in the ischemic storage and in vivo reperfusion groups, thus it may be a theoretical explanation of the reduced vasorelaxation of the grafts.

## Conclusion

In conclusion, the present study investigated the IR injury and impairment of vascular responsiveness induced by in vitro or in vivo IR injury. Vascular models of in vitro reoxygenation and incubation with high dose hypochlorite may be adequate for modelling oxidative stress and so partially the reperfusion injury, but in vivo reperfusion models involve the full scale of damaging effects by restored arterial flow after ischemia. Thus, in vivo methods providing a better understanding of IR mechanism and contribute to develop new preservation solutions. In the present study, mimicking surgical revascularization through aortic transplantation, we clearly demonstrated that the function and the structure of the implanted arterial graft were both severely damaged after short-term storage and warm reperfusion. The impaired endothelium-dependent vasorelaxation and the structural changes in the endothelium may play important roles in the pathogenesis of early graft thrombosis and vasospasm after CABG. However, the functional and structural changes in time could partially recover. Further studies are needed to evaluate the preservation effect of preservation solutions (heparinised blood, Custodiol, UW, Tiprotec). In addition, a deep mechanistic understanding on molecular level of the observed alterations needs to be investigated in other clinically relevant experimental model of bypass grafting.
